# W4Λ: Leveraging Λ Coupled-Cluster for Accurate Computational Thermochemistry Approaches

**DOI:** 10.1021/acs.jpca.3c08158

**Published:** 2024-02-24

**Authors:** Emmanouil Semidalas, Amir Karton, Jan M. L. Martin

**Affiliations:** † Department of Molecular Chemistry and Materials Science, Weizmann Institute of Science, 7610001 Reḥovot, Israel; ‡ School of Science and Technology, University of New England, Armidale, New South Wales 2351, Australia

## Abstract

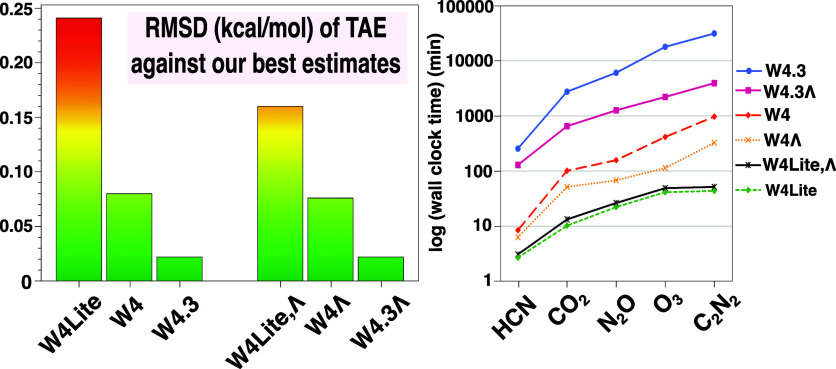

High-accuracy composite wave function methods like Weizmann-4 (W4) theory, high-accuracy extrapolated ab initio thermochemistry (HEAT), and the Feller–Peterson–Dixon (FPD) approach enable sub-kJ/mol accuracy in gas-phase thermochemical properties. Their biggest computational bottleneck is the evaluation of the valence post-CCSD(T) correction term. We demonstrate here, for the W4-17 thermochemistry benchmark and subsets thereof, that the Λ coupled-cluster expansion converges more rapidly and smoothly than the regular coupled-cluster series. By means of CCSDT(Q)_Λ_ and CCSDTQ(5)_Λ_, we can considerably (up to an order of magnitude) accelerate W4- and W4.3-type calculations without loss in accuracy, leading to the W4Λ and W4.3Λ computational thermochemistry protocols.

## 1 Introduction

Chemical thermodynamics and thermochemical kinetics are not just cornerstones of chemistry but arguably its very foundations. As the evaluation of absolute energies of molecules is a Sisyphian task (see Section 5 of ref (1) for a detailed discussion), the most fundamental thermochemical property of a molecule is generally taken to be the heat of formation. While this cannot be directly evaluated computationally, through the heats of formation of the gas-phase atoms, it can be related to the molecular total atomization energy (TAE)—the energy required to break up a molecule into its separate ground-state atoms. This latter quantity—a “cognate” of the heat of formation, if the reader permits a linguistic metaphor—is amenable to computation.

Experimental and theoretical thermochemical techniques have been recently reviewed by Ruscic and Bross.^2^ Nowadays, by far the most reliable source of experimental (or hybrid) reference data are the Active Thermochemical Tables (ATcT) database,^3^ in which a thermochemical network of reaction energies is jointly (rather than sequentially) solved^4,5^ for the unknown heats of formation viz. TAEs and their respective uncertainties.

For small molecules, TAEs can now be evaluated by composite wave function [ab initio] theory (cWFT) approaches to a kJ/mol accuracy or better. Such approaches include Weizmann-4 (W4)^6,7^ and variants (such as W4-F12,^8,9^ W3X-L,^10^ and Wn-P34^11^), the high-accuracy extrapolated ab initio thermochemistry (HEAT) by an international consortium centered on Stanton,^12−15^ the Feller–Peterson–Dixon (FPD) approach,^16,17^ which is less a specific cWFT than a general strategy, and the like. These approaches have been extensively validated against ATcT and other information: see, e.g., Karton^18^ for a recent review.

The “Gold Standard of Quantum Chemistry” (T. H. Dunning, Jr.), CCSD(T) (coupled-cluster with all single and double substitutions with a quasiperturbative correction for triple substitutions),^19,20^ performs much better than it has any right to, owing to a felicitous error compensation amply documented in refs (6, 7, and 12–15) (see Stanton^21^ for a different perspective why this occurs). Post-CCSD(T) valence correlation corrections are the essential component that sets apart W4, HEAT, and the like from lower-accuracy approaches such as the correlation consistent composite approach (ccCA) by the Wilson group,^22^ Gaussian-4 (G4) theory,^23,24^ our own minimally empirical variants of these,^25,26^ or indeed Weizmann-1 (W1) and W2 theory^27^ and their explicitly correlated versions.^28^ In all but the smallest cases, its evaluation is the single greatest “bottleneck” in W4 and HEAT calculations, owing to the extremely steep CPU time scaling of higher-order coupled-cluster approaches. [For fully iterative coupled-cluster up to m-fold connected excitations, the CPU time asymptotically scales as *n*^m^*N*_virt_^*m+2*^ × *N*_iter_, where *N*_iter_ is the number of iterations, *n* is the number of electrons correlated, and *N*_virt_ is the number of virtual (unoccupied) orbitals. For quasiperturbative approximations, the corresponding scaling is *n*^*m*–1^*N*_virt_^*m*+1^*N*_iter_ for the underlying CC(*m* – 1) iterations and *n*^*m*^*N*_virt_^*m*+1^ for the final step].

Hence, any way to significantly reduce their computational cost or make their scaling less steep would extend the applicability of W4- and HEAT-type approaches.

As quasiperturbative triples, (T), proved so successful in ground-state coupled-cluster theory, attempts were then made to add them to excited-state equation-of-motion coupled-cluster theory with all singles and doubles (EOM-CCSD). This led also for the ground state to the so-called Λ coupled-cluster methods,^29−32^ which recently seemed to show promise for computational thermochemistry as well.^33,34^ Moreover, a recent study^35^ on spectroscopic properties of small molecules likewise appeared to show that the Λ quasiperturbative series—CCSD(T)_Λ_, CCSDT(Q)_Λ_, CCSDTQ(5)_Λ_, ...—converges more rapidly than the ordinary quasiperturbative expansion CCSD(T), CCSDT(Q), CCSDTQ(5), ... There was even a tantalizing hint in ref (35) that e.g., CCSDT(Q)_Λ_ might be superior to CCSDTQ owing to a similar error compensation as one sees in CCSD(T) vs CCSDT.

This of course calls for a broader thermochemical exploration: we offer one in the present paper, focusing on the W4-17 benchmark^36^ of 200 first- and second-row molecules, its W4-11 subset^37^ published 6 years earlier, and the latter’s W4-08 subset.^38^ We shall show not only that Λ coupled-cluster indeed accelerates convergence but also that it can be exploited, with no loss in accuracy, for faster and less resource-intensive variants of W4 and W4.3 theory.

## 2 Computational Methods

All calculations were carried out on the Faculty of Chemistry’s HPC facility ChemFarm at the Weizmann Institute of Science.

Geometries of the W4-17 set of molecules, originally optimized at the CCSD(T)/cc-pV(Q+d)Z level with only valence correlation included, were taken from the electronic supporting information (ESI) of the W4-17 paper^36^ and used as-is, without further optimization.

Most of the post-CCSD(T) electronic structure calculations, and all of the post-CCSDTQ calculations, were carried out using the arbitrary-order coupled-cluster code^39−42^ in the MRCC program system of Kállay and co-workers.^43^ The specific levels of theory considered include CCSDT,^44^ CCSDT[Q],^45^ CCSDT(Q),^39^ CCSDT(Q)_A_,^41^ CCSDT(Q)_B_,^41^ CCSDTQ,^46^ CCSDTQ(5),^41^ and CCSDTQ5.^47^

Coupled-cluster jobs were run in a “sequential restart” fashion where, e.g., CCSDT takes initial *T*_1_ and *T*_2_ amplitudes from the converged CCSD calculation, CCSDTQ in turn uses the converged CCSDT amplitudes as initial guesses for the *T*_1_, *T*_2_, and *T*_3_ amplitudes, and so forth. For the open-shell species, unrestricted Hartree–Fock references were used throughout, except that higher-order triple excitation contributions, *T*_3_ – (T), were also evaluated restricted open-shell in semicanonical orbitals as per the original W4 protocol.

The most demanding CCSDT, CCSDT(Q), CCSDT(Q)_Λ_, and CCSDTQ calculations were carried out using a prerelease version of the NCC program developed by Matthews and co-workers as part of CFOUR.^48^

Basis sets considered are the cc-pVnZ basis sets of Dunning and co-workers^49,50^ or truncations thereof. The abbreviated notation we use for truncated basis sets is probably best illustrated by example: VDZ(p,s) refers to cc-pVDZ truncated at p functions for nonhydrogen and at s functions for hydrogen, VDZ(d,s) refers to the untruncated cc-pVDZ basis set on nonhydrogen atoms, and the p polarization functions on hydrogen are removed.

It is well-known (e.g., refs (51 and 52)) that for second-row atoms in high oxidation states, tight (i.e., high-exponent) d functions are energetically highly important at the CCSD(T), or even the Hartree–Fock (!), level. (This was ultimately rationalized^53^ chemically as back-donation from chalcogen and halogen lone pairs into the vacant 3d orbital, which drops closer to the valence orbitals in energy as the oxidation state increases. A similar phenomenon involving tight f functions and vacant 4f and 5f orbitals exists in heavy p-block compounds.^54^) However, do tight d functions significantly affect post-CCSD(T) contributions? One of us^55^ considered this question and found the total contribution to be quite modest and to largely cancel between higher-order triples and connected quadruples.

## 3 Results and Discussion

### 3.1 Higher-Order Connected Sextuples

Table 1 summarizes our results using coupled-cluster methods for the molecular sets considered in this study.

**Table 1:** Overview of Post-CCSD(T) Methods and Basis Sets Used for Molecules in the W4-17 Database

method	basis set	#species	data set^a^
CCSDT	VDZ(p,s) through		
	VQZ(f,d)	200	W4-17
	VQZ(g,d)	199	W4-17 except *n*-pentane
	V5Z(h,f)	65	“W4.3” subset
CCSDT(Q)	VDZ(p,s)	200	W4-17
	VDZ(d,s)	200	W4-17
	VTZ(d,p)	199	W4-17 except C_2_Cl_6_
	VTZ(f,p)	198	W4-17 except C_2_X_6_ (X = F, Cl)
	VTZ(f,d)	197	W4-17 except n-pentane, C_2_X_6_ (X = F, Cl)
	VQZ(d,p)	137	W4-11
	VQZ(f,d)	132	W4-11: excluding 5 species^b^
	VQZ(g,d)	122	W4-11: excluding 12 species, W4-08: without 3^c^
	V5Z(h,f)	59	“W4.3” subset excluding 6 species^d^
CCSDT(Q)_Λ_	VDZ(p,s)	200	W4-17
	VDZ(d,s)	200	W4-17
	VTZ(d,p)	188	W4-17: excluding 12 species^e^
	VTZ(f,p)	157	W4-11 plus 20 species from W4-17
	VQZ(g,d)	122	W4-11: excluding 12 species, W4-08: without 3^f^
CCSDTQ	VDZ(p,s)	200	W4-17
	VDZ(d,s)	184	W4-17: excluding 16 species
	VTZ(d,p)	134	W4-08 except AlF_3_, AlCl_3_, BF_3_, O_2_F_2_, S_4_, SO_3_ plus 25 species from W4-11 and 19 from W4-17
	VTZ(f,p)	65	“W4.3” subset
	VQZ(g,d)	50	“W4.3” subset except 15 species^g^
CCSDTQ(5)	VDZ(p,s)	193	W4-17 except 7 species^h^
	VDZ(d,s)	165	W4-17 except 32 species; W4-11 except CH_3_COOH, CF_4_, and SiF_4_
	VTZ(f,p)	53	“W4.3” subset except 12 species
CCSDTQ(5)_Λ_	VDZ(p,s)	193	W4-17 except 7 species^h^
	VDZ(d,s)	163	W4-17 except 34 species; W4-11 except CH_3_COOH, CF_4_, and SiF_4_
	VTZ(f,p)	53	“W4.3” subset except 12 species
CCSDTQ5	VDZ(p,s)	96	W4-08
	VDZ(d,s)	65	“W4.3” subset
CCSDTQ5(6)	VDZ(p,s)	95	W4-08 except O_2_F_2_
CCSDTQ5(6)_Λ_	VDZ(p,s)	95	W4-08 except O_2_F_2_
CCSDTQ56	VDZ(p,s)	88	W4-08 except BF_3_, C_2_N_2_, O_2_F_2_, AlF_3_, P_4_, SO_3_, S_4_, AlCl_3_
core–valence post-CCSD(T) correlation contributions			
CCSDT – CCSD(T)	CVTZ(f,p)	136	W4-11except *cis*-HOOO
CCSDT(Q) – CCSDT	CVTZ(f,p)	118	W4-08: excluding 8 species; W4-11: except 11 species^i^

a W4-17, W4-11, and W4-08 data sets include 200, 137, and 96 species, respectively; the “W4.3” subset for which W4.3 results were obtained in earlier work contains 65 species.

b Without acetic acid, ethanol, CF_4_, C_2_H_5_F, and propane.

c Excluding from W4-08: FO_2_, O_2_F_2_, and ClOO; excluding from W4-11: acetaldehyde, formic and acetic acids, ethanol, glyoxal, *cis*-HOOO, CF_4_, SiF_4_, C_2_H_5_F, propane, propene, and propyne.

d Without CH_2_CH, CH_2_NH, NO_2_, N_2_O, H_2_O_2_, and F_2_O.

e Without *n*-pentane, benzene, C_2_X_6_ (X = F, Cl), PF_5_, SF_6_, *cis*-C_2_F_2_Cl_2_, cyclopentadiene, beta-lactim, ClF_5_, *n*-butane, and allyl.

f Excluding from W4-11: acetaldehyde, formic and acetic acids, ethanol, glyoxal, *cis*-HOOO, CF_4_, SiF_4_, C_2_H_5_F, propane, propene, and propyne; excluding from W4-08: FO_2_, O_2_F_2_, and ClOO.

g Without CH_2_C, CH_2_CH, C_2_H_4_, CH_2_NH, HCO, H_2_CO, CO_2_, NO_2_, N_2_O, O_3_, HOO, H_2_O_2_, F_2_O, SSH, and HOF.

h Without *n*-pentane, C_2_X_6_ (X = F, Cl), cyclopentadiene, silole, beta-lactim, and ClF_5_.

i Excluding from W4-08: AlCl_3_, AlF_3_, BF_3_, S_4_, S_3_, CS_2_, SO_3_, and P_4_; excluding from W4-11: allene, propyne, propane, F_2_CO, C_2_F_2_, C_2_H_5_F, SiF_4_, CF_4_, *cis*-HOOO, glyoxal, and acetic acid.

**Figure 1:**
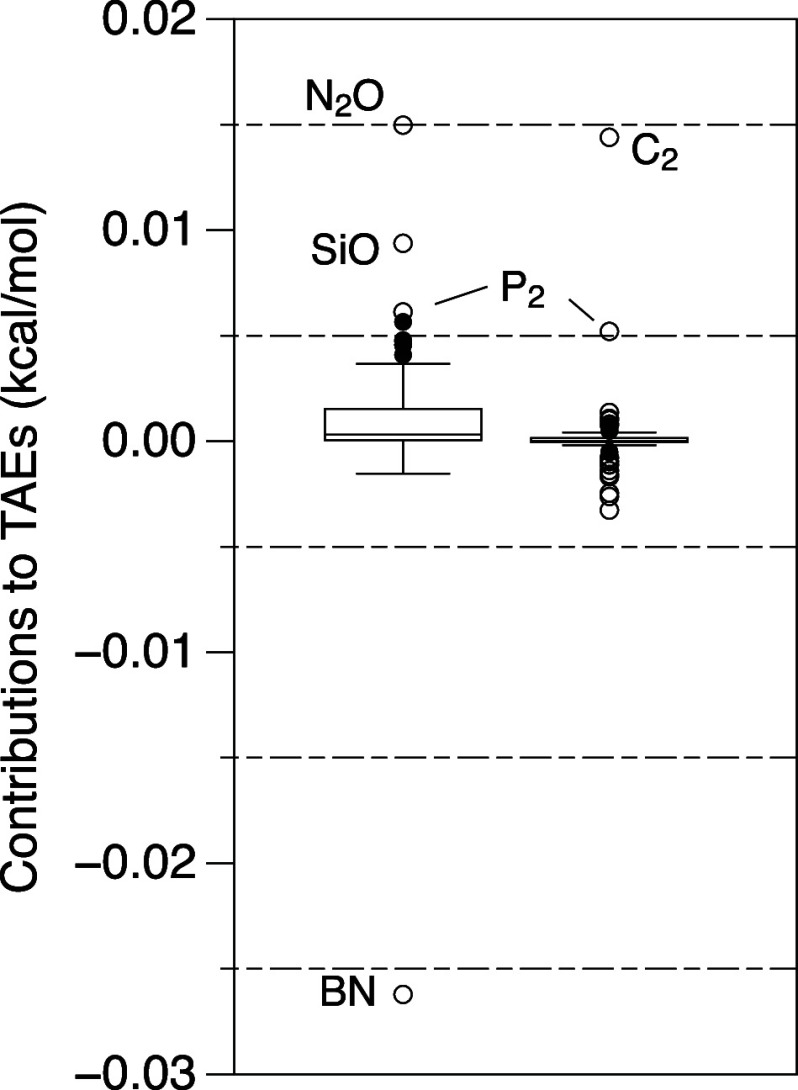
Box-and-whiskers plots of  (on the left) and  (on the right) using the VDZ(p,s) basis set for the W4-08 data set (excluding BF_3_, C_2_N_2_, O_2_F_2_, AlF_3_, P_4_, SO_3_, S_4_, and AlCl_3_). In this and subsequent box plots, the box boundaries represent the 25th and 75th percentile of the data, and the whiskers extend to the last point within 1.5 times the interquartile range (IQR) from the box, following the standard Tukey definition. Outliers are shown as filled circles if located more than 1.5 IQR from the box edge, while extreme outliers are represented by open circles when positioned more than 3 IQR from the box edge.

The smallest contribution we will consider here are the higher-order connected sextuple excitations, CCSDTQ56 – CCSDTQ5(6) or  for short, and CCSDTQ56 – CCSDTQ5(6)_Λ_ or  for short. A box-and-whiskers plot of these contributions is given in Figure 1. It can be seen there that  has an extremely narrow spread, and that the largest outlier by far is C_2_ at just 0.015 kcal/mol. We can hence consider CCSDTQ5(6)_Λ_ to be essentially equivalent to CCSDTQ56 in quality.

For CCSDTQ56 – CCSDTQ5(6), the spread is still very narrow, but now we have some small positive and negative outliers, BN −0.026, N_2_O +0.015, SiO +0.009, P_2_ +0.006 kcal/mol.

Since these tiny contributions are much smaller than the basis set incompleteness error in the larger components (see below), we feel justified in neglecting higher-order connected sextuples altogether.

### 3.2 CCSDTQ5(6)_Λ_ – CCSDTQ(5)_Λ_

**Figure 2:**
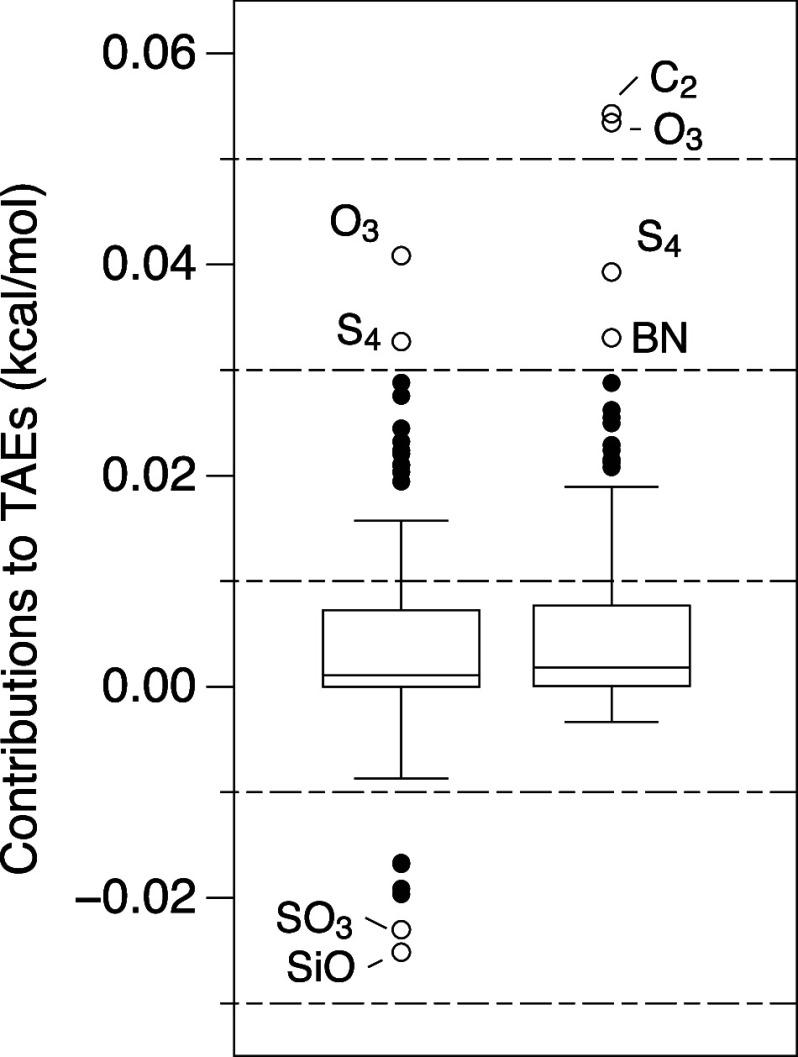
Box-and-whiskers plots of (6)_Λ_ – (5)_Λ_ (on the left) and (6)_Λ_ – *T*_5_ (on the right) using the VDZ(p,s) basis set for the W4-08 data set.

The quasiperturbative sextuple contributions were evaluated only for the VDZ(p,s) basis set. A box plot can be seen in Figure 2. Whiskers are at +0.02 and −0.01 kcal/mol, around a median of basically 0.00 kcal/mol. The largest positive and negative outliers are +0.04 and −0.03 kcal/mol, respectively. In almost all situations, this contribution can be safely neglected.

If we used full iterative CCSDTQ56 – CCSDTQ5 instead (at massively greater computational expense), we would get binding contributions throughout (small as they might be), topping out at 0.069 kcal/mol for C_2_.

We argue that this contribution can be omitted in all but the most accurate work.

This is not the case for CCSDTQ5(6) – CCSDTQ(5), given the well-known deficiencies of CCSDTQ(5).^7,40^

### 3.3 CCSDTQ(5)_Λ_ – CCSDT(Q)_Λ_

We now move on to the Λ connected quintuples contribution, CCSDTQ(5)_Λ_ – CCSDT(Q)_Λ_. The largest basis set for which we were able to evaluate even a subset (53 species) was VTZ(f,p).

**Figure 3:**
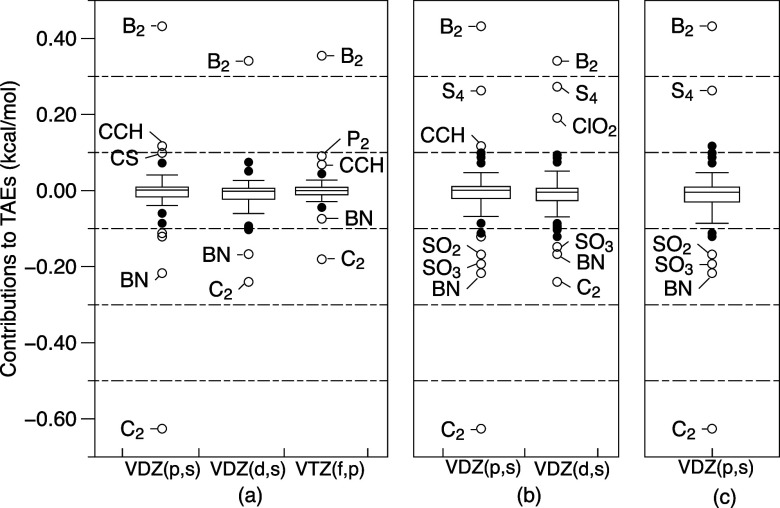
Box-and-whiskers plots of contributions to TAEs of (5)_Λ_ – (Q)_Λ_ terms for (a) “W4.3” subset, (b) W4-08, and (c) W4-11 data sets.

As can be seen in Figure 3, the box is centered near 0.0 while the whiskers are ±0.04 kcal/mol in the largest basis set, VTZ(f,p). For the “W4.3” subset of 65 species, the root-mean square deviation (rmsd) between VDZ(p,s) and VTZ(f,p) is 0.06 kcal/mol, while that between VDZ(d,s) and VTZ(f,p) shrinks to just 0.02 kcal/mol.

For the smallest basis set, VDZ(p,s), there are sizable outliers, at −0.63 kcal/mol (for C_2_) and +0.43 kcal/mol (for B_2_). These shrink to −0.24 and +0.34 kcal/mol, respectively, for VDZ(d,s), i.e., upon expanding the nonhydrogen basis sets from double-ζ to polarized double-ζ.

Obviously, for small “troublemakers” like C_2_, BN, and B_2_, switching to a basis set larger than VDZ(p,s) is not an issue at all.

In contrast, for CCSDTQ5 versus CCSDTQ, most contributions are positive. VDZ(p,s) has a box at about 0.08 kcal/mol with whiskers spanning 0.2 kcal/mol (outliers at 0.4 kcal/mol) while VDZ(d,s) has a smaller box (0.06 kcal/mol), and its whiskers span 0.14 kcal/mol (outliers up to 0.42 kcal/mol) (see Figure S1 in Supporting Information).

Therefore, aside from the much lower cost of CCSDTQ(5)_Λ_ compared to CCSDTQ5, the CCSDTQ(5)_Λ_ – CCSDT(Q)_Λ_ contribution is close enough to zero that “in a pinch” it can be omitted altogether.

Nevertheless, let us also consider the quintuples contributions relative to CCSDTQ. For the 53 VTZ(f,p) species, the rms CCSDTQ(5)_Λ_ – CCSDTQ is just 0.08 kcal/mol with the VTZ(f,p) basis set. For the same contribution, the rmsd between VDZ(p,s) and VTZ(f,p) basis sets is just 0.02 kcal/mol, while that between VDZ(d,s) and VTZ(f,p) shrinks to just 0.01 kcal/mol. (Box plots of CCSDTQ(5)_Λ_ – CCSDTQ contributions to TAEs are shown in Figure S2 of Supporting Information).

What about (5) vs (5)_Λ_ contributions compared to full iterative CCSDTQ5 – CCSDTQ? We have [CCSDTQ5 – CCSDTQ]/VDZ(d,s), or if you like, /VDZ(d,s) available for the “W4.3” subset of molecules, with which the rmsd of (5) is 0.05 kcal/mol, compared to just 0.01 kcal/mol for (5)_Λ_. For the small VDZ(p,s) basis set, the rmsd between (5) and  is 0.04 kcal/mol, compared to 0.01 kcal/mol between (5)_Λ_ and . We believe that this adequately shows the superiority of (5)_Λ_.

### 3.4 Higher-Order Quadruples

The relevant statistics for the higher-order quadruples,  and , are given in Table 2, complemented by two box plots as shown in Figures S3 and S4 while the outliers are listed in Table S1 in Supporting Information. It can be seen in Table 2 that complete neglect beyond CCSDT(Q) would cause an error of 0.255 kcal/mol, but only 0.111 kcal/mol beyond CCSDT(Q)_Λ_. Introducing a low-cost CCSDTQ/VDZ(p,s) calculation would reduce rmsd to 0.094 kcal/mol for  but 0.062 kcal/mol for —the latter is small enough that one is tempted to substitute a single CCSDTQ(5)_Λ_/cc-pVDZ(p,s) calculation for the W4 theory combination of CCSDTQ/cc-pVDZ and CCSDTQ5/cc-pVDZ(p,s).

**Table 2:** rmsd and Mean Signed Deviations (MSDs) in kcal/mol of  and  Terms for the “W4.3” Subset

basis set	a		b	
	rmsd	MSD	rmsd	MSD
neglecting	0.255	0.109	0.111	0.044
VDZ(p,s)	0.094	0.039	0.062	0.007
VDZ(d,s)	0.030	0.011	0.014	0.001
VTZ(d,p)	0.015	0.003	0.010	0.001
VTZ(f,p)	0.012	0.006	0.007	0.005
VQZ(g,d)	REF	REF	REF	REF

a /VQZ(g,d) is used as reference.

b /VQZ(g,d) is used as reference. A total of 50 data points are used in all comparisons.

Similarly, with the cc-pVDZ(d,s) basis set, we have rmsd = 0.030 kcal/mol for  , but just 0.014 kcal/mol for . With , one needs to escalate to VTZ(f,p) (like in W4.3 theory) to achieve a comparable rmsd = 0.012 kcal/mol.

### 3.5 (Q)_Λ_ – (Q)

The difference between ordinary parenthetical quadruples and their Λ counterpart approaches zero for systems dominated by dynamical correlation but becomes quite significant when there is strong static correlation. [In ref (6), we defined the %TAE[(T)] diagnostic, the percentage of the CCSD(T) TAE that is due to connected triples, as a “pragmatic” diagnostic for static correlation (see also refs (56 and 57) for other diagnostics)]. Between %TAE[(T)] and %TAE[(Q)_Λ_ – (Q)], and upon eliminating the irksome BN diatomic, the coefficient of determination *R*^2^ for 160 closed-shell species is 0.7164 with the cc-pVDZ(d,s) basis set, which increases to 0.7407 upon additionally eliminating ClF_5_. While this is not something one would want to substitute for an actual evaluation, it does indicate a relationship between the two quantities.

Compared to the largest basis set for which we have sufficient data points available, namely, VQZ(g,d), the rmsd is 0.066 kcal/mol for VDZ(p,s) but drops to 0.027 kcal/mol for VDZ(d,s) and to 0.01 kcal/mol or less for a VTZ basis set.

In W4 theory, we combined^6^ CCSDT(Q)/VTZ(f,d) with [CCSDTQ – CCSDT(Q)]/VDZ(d,p). If, for the sake of argument, we split up the latter term into [CCSDT(Q)_Λ_ – CCSDT(Q)]/VDZ(d,p) plus [CCSDTQ – CCSDT(Q)_Λ_]/VDZ(d,p), then based on the previous section, we could prune the basis set for the second step to VDZ(p,s) and greatly reduce computational expense.

Continuing this line of argument, in the W4.3 and W4.4 theories, CCSDT(Q)/V{T,Q}Z extrapolation is combined with [CCSDTQ-CCSDT(Q)]/VTZ(f,d). If we again partition the latter term into a relatively cheap [CCSDT(Q)_Λ_ – CCSDT(Q)]/VTZ(f,p) and a very expensive [CCSDTQ – CCSDT(Q)_Λ_]/VTZ(f,p) step, we could again take down the basis set for the latter to VDZ(d,s).

Why not do the largest basis set (Q)_Λ_ to begin with? The extra computational expense of evaluating the “left eigenvector” is of course one factor but not the main one: in practice, we find the additional memory requirements to be a greater impediment for large molecules and basis sets.

### 3.6 Parenthetical Connected Quadruples (Q)

In W4lite and W4 theory, the (Q) contribution is included via scaling, as it was shown^6^ that extrapolation of (Q) from too small basis sets yields erratic results for highly polar molecules like H_2_O and HF.

For calibration, we used V{Q,5}Z extrapolation for 58 species (“W4.3” subset minus CH_2_CH, CH_2_NH, NO_2_, N_2_O, H_2_O_2_, and F_2_O). Minimizing rmsd against that (see Table 3), we find the cheapest option that still has a tolerably small rmsd to be VDZ(d,s) scaled by 1.227, close enough to the 5:4 used in the past for  in W3 theory.^58^ The error drops to 0.082 kcal/mol with 1.17 × VTZ(d,p) and to 0.038 kcal/mol with 1.099 × VTZ(f,p), only semantically different from 1.1 × VTZ used in W4 theory. This is roughly half the rmsd of V{D,T}Z extrapolation, at rmsd = 0.074 kcal/mol (Figure S5).

**Table 3:** rms (kcal/mol) of CCSDT(Q)-CCSDT Errors for TAEs in the “W4.3” Subset

basis set	rmsd
VDZ(p,s)	0.413
VDZ(d,s)	0.249
VTZ(d,p)	0.183
VTZ(f,p)	0.109
VTZ(f,d)	0.108
VQZ(f,d)	0.058
VQZ(d,p)	0.149
VQZ(g,d)	0.044
V5Z(h,f)	0.021
1.227 × VDZ(d,s)^a^	0.139
1.170 × VTZ(d,p)^a^	0.082
1.099 × VTZ(f,p)^a^	0.038
V{D(d,s),T(f,p)}Z^b^	0.074
V{T(d,p),Q(f,d)}Z^a^	0.010
V{T(f,p),Q(g,d)}Z^b^	0.006
(Q)/V{Q(g,d),5(h,f)}Z	REF

a Scaling factor obtained from rmsd minimization.

b Extrapolation exponent *a* = 2.85 for V{D,T}Z and *a* = 3.25 for V{T,Q}Z; no change occurs with Karton’s extrapolation exponents (*a* = 2.9968 and *a* = 3.3831) from Table 5 in ref (59). A total of 58 species are included in all comparisons.

V{T,Q}Z as used for W4.3 theory in ref (6), at rmsd = 0.006 kcal/mol is essentially as accurate as the reference. Importantly, however, deleting the highest angular momentum in both basis sets is found to cause only negligible further loss of accuracy, to rmsd = 0.01 kcal/mol. This offers an attractive way to reduce the cost of W4.3 calculations (see below).

### 3.7 Higher-Order Connected Triples, CCSDT – CCSD(T)

For a subset of 65 molecules within W4-08—the so-called “W4.3” subset for which we were able to perform W4.3 calculations in refs (36 and 37)—we managed to carry out CCSDT/cc-pV5Z calculations, and hence, we used CCSD(T)/V{Q,5}Z extrapolation as the reference value. These limits are apparently not very sensitive to the extrapolation exponent, as there is just 0.007 kcal/mol rms difference between values obtained using a fixed 3.0 and Karton’s optimized value^59^ of 2.7342. That means that the reference we are using is pretty insensitive to details of the extrapolation procedure; hence it makes sense to calibrate the  – (*T*) higher-order connected triples contribution to TAE by comparison with [CCSDT – CCSD(T)]/V{Q,5}Z extrapolations.

**Figure 4:**
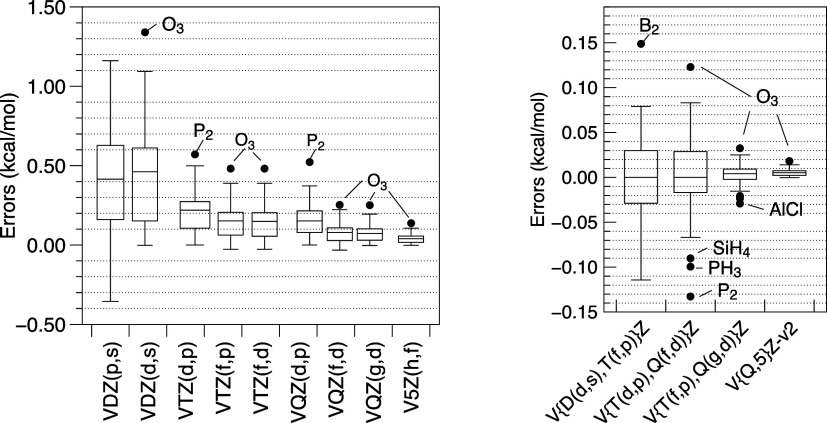
Errors for  – (T) terms vs  – (T)/V{Q(g,d),5(h,f)}Z for the “W4.3” subset with 65 molecules.

The rms  – (T) contribution is 0.684 kcal/mol. It is obvious from Figure 4 and Table 4 that untruncated and truncated cc-pVDZ basis sets are barely better than doing nothing, with rms errors over 0.5 kcal/mol. A cc-pVTZ basis set recovers a more respectable chunk, but still leaves 0.18 kcal/mol rmsd, which increases to 0.25 kcal/mol if the f functions are omitted. In order to get below 0.1 kcal/mol without extrapolation, at least a cc-pVQZ basis set is required, although the g function can apparently be safely omitted. V5Z reaches 0.05 kcal/mol.

**Table 4:** rmsd  against  – (T)/V{Q,5}Z (kcal/mol) for the “W4.3” Subset

basis set	rmsd^a^
neglecting	0.684
VDZ(p,s)	0.554
VDZ(d,s)	0.545
VTZ(d,p)	0.248
VTZ(f,d)	0.179
VTZ(f,p)	0.181
VQZ(f,d)	0.094
VQZ(g,d)	0.090
VQZ(d,p)	0.194
V5Z(h,f)	0.049

Schwenke coefficient *A*_L_^b^		rmsd^a^
V{D,T}Z	0.503	0.041
V{D,T}Z	0.498^c^	0.041
V{D,T}Z	0.570	0.052
V{T,Q}Z	0.950	0.012
V{T(d,p),Q(f,d)}Z	0.528^d^	0.042
V{Q,5}Z-v2	1.049	0.007
V{Q,5}Z	1.210	REF

a All 65 molecules of the “W4.3” subset are considered for all comparisons.

b The energy is extrapolated with the Schwenke^60^ two-point formula *E*_∞_ = *E* + *A*_L_(*E*(L) – *E*(L – 1)).

c Exponent taken from Karton.^59^

d Obtained from rmsd minimization.

Extrapolation from cc-pV{D,T}Z, as practiced in W4 theory,^6^ brings down rmsd to just 0.041 kcal/mol with the exponent 2.7174 optimized by Karton:^59^ if one substitutes the 2.5 recommended in refs (6 and 7), rmsd slightly increases to 0.052 kcal/mol. For cc-pV{T,Q}Z, Karton’s exponent is functionally equivalent to 2.5, and we obtain rmsd = 0.012 kcal/mol—that level as used in W4.3^6^ and W4.4^7^ can credibly be used as a reference. Alas, unlike for (Q), removal of the top angular momenta of both basis sets increases the error to the same as cc-pV{D,T}Z, presumably primarily because of the impact on cc-pVTZ.

### 3.8 Post-CCSD(T) Core–Valence Contributions

Core–valence  is required for W4.2 and W4.3 theory^6^ while core–valence (Q) corrections enter in W4.4 theory.^7^ In the original papers,^6,7^ we employed the cc-pCVTZ basis set;^61^ in the present work, we employed the combination of cc-pCVTZ on nonhydrogen atoms and cc-pVTZ(no d) on hydrogen—abbreviated, CVTZ(f,p). Full CCSDT proved feasible for all of W4-11 (except for *cis*-HOOO, owing to an SCF convergence issue) while (Q) was feasible for W4-08 minus eight species (seven of which contain multiple second-row atoms with their [Ne] cores) and for the additional W4-11 species minus 10 larger first-row species and SiF_4_.

The rms core–valence  contribution was 0.046 kcal/mol and the rmsd core–valence (Q) term was 0.037 kcal/mol—larger than the remaining errors in the valence post-CCSD(T) part of W4.3 theory and hence not negligible.

W4.2 theory is identical to W4 theory except for the core–valence  term. As discussed in ref (6), it removes the dependence on the specific definition of frozen-core CCSD(T) for restricted open-shell references (semicanonicalization before transform as in Gaussian,^62^ MRCC, and CFOUR versus transform before semicanonicalization as in MOLPRO^63^).

### 3.9 Composite Approaches

First, let us concentrate on W4 itself. In composite A, we retain the  component from original W4, except that we remove the top angular momentum from hydrogen: CCSDT/{VDZ(d,s),VTZ(f,p)}. Next, we add (Q)/VTZ(f,p) and scale the contribution by 1.11. Then, we add [(Q)_Λ_ – (Q)]/VDZ(d,s) and CCSDTQ(5)_Λ_/VDZ(p,s) – CCSDT(Q)_Λ_/VDZ(p,s). In effect, we replace the CCSDTQ/VDZ(d,s) and CCSDTQ5/VDZ(p,s) steps with a single CCSDTQ(5)_Λ_/VDZ(p,s) step.

Composite A is feasible for the entire W4-17 data set except for seven species where the quintuples present an obstacle. As they are expected to be of minor importance in these species, one can substitute CCSDTQ/VDZ(p,s) as a fallback option, leaving us with a complete W4-17 set. By way of illustration, for NCCN (dicyanogen) on 16 cores of an Intel Ice Lake server at 2.20 GHz with 768 GB RAM and local SSD, the CCSDT(Q), CCSDTQ, and CCSDTQ5 steps of standard W4 theory take 16.2 h wall time, compared to 5.4 h for the corresponding steps in Composite A, making it 3 times faster. We thus renamed composite A to W4Λ. The rmsd between this W4Λ and standard W4 is 0.066 kcal/mol for the post-CCSD(T) contributions to TAEs of the W4-08 data set.

Now, let us consider W4.3. In composite B, we retain the  component from original W4, except that we remove the top angular momentum from hydrogen: CCSDT/{VTZ(f,p),VQZ(g,d)}. The (Q) we extrapolate from (Q)/{VTZ(d,p),VQZ(f,d)}. Then, we add [(Q)_Λ_ – (Q)]/VTZ(f,p) and CCSDTQ(5)_Λ_/VDZ(d,s) – CCSDT(Q)_Λ_/VDZ(d,s).

This composite B is feasible for nearly all of the W4-11 set; in conjunction with the CCSDT/CVTZ(f,p) core–valence contribution, we dub it here W4.3Λ. The rmsd between W4.3Λ and standard W4.3 is only 0.019 kcal/mol for the TAEs of 65 species in the “W4.3” data set.

In composite C, we add a CCSDTQ5(6)_Λ_/VDZ(p,s) – CCSDTQ(5)_Λ_/VDZ(p,s) component to composite B. Together with the CCSDT(Q)/CVTZ(f,p) core–valence contribution, we propose this as W4.4Λ.

What about lower-cost options? A form of W4lite,Λ can be created by scaling (Q)_Λ_/VDZ(d,s) by an empirical scale factor obtained by minimizing the rmsd from W4.3Λ: we thus find a scale factor of 1.249 (in practice, 5:4) and rmsd = 0.149 kcal/mol.

At a reviewer’s request, the final leaf of the ESI workbook compares the original W4-17 atomization energies at W4 and W4.*n* levels with their Λ counterparts. As the differences between them are essentially confined to the post-CCSDT terms (leaving aside the small differences in *T*_3_ – (T) owing to the present omission of the highest angular momentum in the hydrogen basis set), differences between the two are very small as expected, with an IQR = 0.04 kcal/mol. The one glaring exception is FOOF, where the discrepancy reached nearly 1 kcal/mol. Upon close scrutiny, we were able to identify an error in the published W4-11 and W4-17 values for that molecule and to trace those to a CCSDT(Q)/cc-pVTZ calculation where a malfunctioning restart from saved amplitudes (back in 2007) yielded an erroneous total energy of −349.4117061 *E*_h_ rather than the correct value of −349.4131206 *E*_h_. As a result, the published^36^ TAE_e_ value of 151.00 kcal/mol should read 151.89 kcal/mol, and its TAE_0_ counterpart of 146.00 should read 146.89 kcal/mol instead. In addition, in Table S2 of the article mentioned above, the T_4_ contribution for FOOF should read 3.18 rather than 2.29 kcal/mol.

### 3.10 Timing Comparison of Composite Approaches

The total electronic energy of a W4-type composite approach is the sum of CCSD(T) energy near the basis set limit and various post-CCSD(T) terms, detailed in previous sections. As for all but the smallest molecules, the cost of the post-CCSD(T) contributions dwarfs that of the CCSD(T)/CBS step, our timing comparison can focus exclusively on the former. For example, three separate single-point energy calculations are needed for W4: (a) CCSDTQ5/VDZ(p,s); (b) CCSDTQ/VDZ(d,s); and (c) CCSDT(Q)/VTZ(f,p). The /V{D,T}Z term is a byproduct of parts (b) and (c). Note that CCSDT(Q), CCSDTQ5, and CCSDTQ56 scale with system size as *N*^9^, *N*^12^, and *N*^14^, respectively.^18^

**Figure 5:**
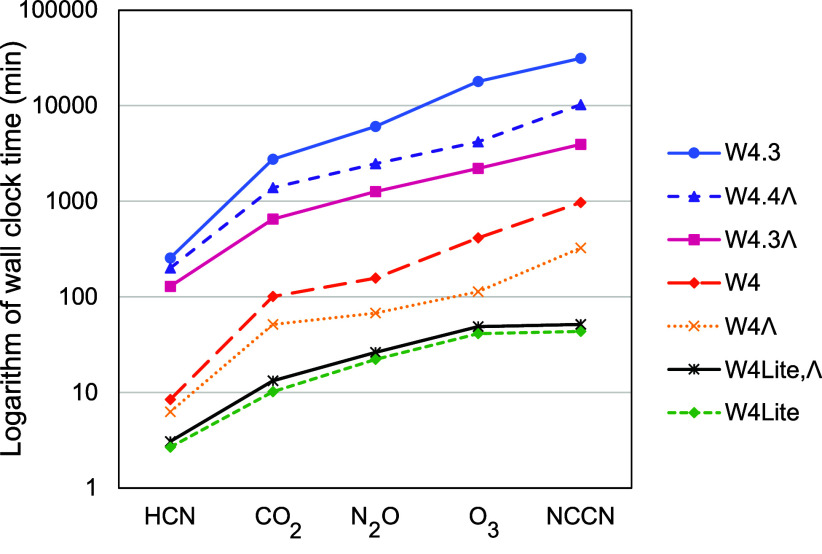
Wall clock time for calculating the post-CCSD(T) terms of various composite schemes for five molecules in the W4-11 data set. The *y* axis is in the logarithmic scale.

Figure 5 shows total wall clock times (note the logarithmic *y* axis) for calculating the post-CCSD(T) terms of several composite approaches across five species in the W4-08 data set. Calculations were performed on 16 cores and in 340 GB of RAM on otherwise empty nodes with dual 26-core Intel Ice Lake CPUs at 2.2 GHz, 7.0 TB of local solid state disk, and 768 GB of RAM. Wall clock times and percentages of time elapsed per step are provided in Tables S2 and S4 of Supporting Information.

Λ-based composites, especially for larger molecules, exhibit significant speedups over non-Λ counterparts. For cyanogen, standard W4 takes 16.3 h, while W4Λ only requires one-third as much (5.4 h); W4.3 requires 522.3 h, but W4.3Λ only one-eighth thereof (65.6 h). Both W4lite and W4lite,Λ are almost of equivalent cost for the molecules considered. As can be seen from Table S4, for W4 the highest-order coupled-cluster step, CCSDTQ56/VDZ(p,s), will dominate the CPU time as the molecules grow larger; in contrast, for W4Λ, the CCSDT(Q)/VTZ step remains the dominant one. If the calculation is carried out wholly using MRCC, then the (Q) step benefits from almost perfect parallelism, which offers a further way to speed up the W4Λ calculation if enough cores are available. If, on the other hand, the CCSDT(Q) step is carried out using the very fast NCC module in CFOUR, then this will further favor W4Λ over W4 as the dominant iterative quintuples step in the latter is not amenable to NCC at present. For much larger systems, the total cost of canonical calculations would of course become intractable, but their CPU time scaling can be nearly linearized by substituting localized natural orbital coupled-cluster, LNO-CCSDT(Q),^64−66^ for conventional CCSDT(Q). The performance of the latter is presently being investigated in our laboratory.

## 4 Conclusions

Using the W4-17 data set (and the W4-11 and W4-08 subsets thereof) as our “proving ground”, we have reconsidered post-CCSD(T) corrections in computational thermochemistry in light of Λ coupled-cluster methods. It is apparent that the Λ approach converges more rapidly and smoothly with the substitution levels. Our findings corroborate our earlier conjecture^35^ that the coupled-cluster series has two more “sweet spots” (performance-price optima) beyond CCSD(T), namely, CCSDT(Q)_Λ_ and CCSDTQ(5)_Λ_. These findings are then exploited to drastically reduce the computational requirements of the W4 and W4.3 computational thermochemistry protocols; we denote the modified versions W4Λ and W4.3Λ, respectively.

## Data Availability

This material is also freely available from the Figshare repository at http://doi.org/10.6084/m9.figshare.24806913. Additional data can be obtained from the authors upon reasonable request.

## Supporting Information Available

Total energies, TAE contributions, and statistics (ZIP)
Box plots of CCSDTQ5 – CCSDTQ contributions to TAEs, CCSDTQ(5)_Λ_ – CCSDTQ contributions, CCSDTQ – CCSDT(Q) and CCSDTQ – CCSDT(Q)_Λ_ basis set convergence, CCSDT(Q) – CCSDT differences; relative errors of  – (Q) and  – (Q)_Λ_ compared to [ – (Q)]/VQZ(g,d) and [ – (Q)_Λ_]/VQZ(g,d); wall clock times of composite approaches for selected species; wall clock times per post-CCSD(T) step of each composite approach; refs (43, 62, 63) with full author lists (PDF)
