# Supplementary material for: W4Λ: Leveraging Λ Coupled-Cluster for Accurate Computational Thermochemistry Approaches
Source: J Phys Chem A. 2024 Feb 24;128(9):1715–24. doi: 10.1021/acs.jpca.3c08158 (PMC10926103; doi:10.1021/acs.jpca.3c08158)
Supplement: Supplementary file 2 — jp3c08158_si_002.pdf [file jp3c08158_si_002.pdf]

Supporting Information: W4Λ: Leveraging Λ Coupled Cluster for Accurate Computational Thermochemistry Approaches

Emmanouil Semidalas[1], Amir Karton[2], and Jan M. L. Martin[1,a)]

[1]*Dept. of Molecular Chemistry and Materials Science, Weizmann Institute of Science, 7610001 Reḥovot, Israel.*
[2]*School of Science and Technology, University of New England, Armidale, NSW 2351, Australia.*

E-mail: gershom@weizmann.ac.il

[a)]URL: http://www.compchem.me

Table of Contents

Figure S1. Box plots of CCSDTQ5 – CCSDTQ contributions to TAEs S1

Figure S2. Box plots of CCSDTQ(5)$_{Λ}$ – CCSDTQ contributions to TAEs S1

Figure S3. Box plots of CCSDTQ – CCSDT(Q) errors for TAEs... S2

Figure S4. Box plots of CCSDTQ – CCSDT(Q)$_{Λ}$ errors for TAEs.. S2

Figure S5. Box plots of CCSDT(Q) – CCSDT errors for TAEs relative to [CCSDT(Q) – CCSDT]/V{Q(g,d),5(h,f)}Z for the 'W4.3' subset. S3

Table S1: Relative errors (kcal/mol) of [$\hat{T}_4$ – (Q)] and [$\hat{T}_4$ – (Q)$_{Λ}$] compared to [$\hat{T}_4$ – (Q)]/VQZ(g,d) and [$\hat{T}_4$ – (Q)$_{Λ}$]/VQZ(g,d), respectively, for selected species of the 'W4.3' subset..................... S4

Table S2. Wall clock times (min) of composite approaches on 16 physical cores of a node equipped with dual 26-core Intel Ice Lake CPUs (2.20 GHz) for selected species of the W4-11 dataset. .. S4

Table S3. Wall clock times (min) per post-CCSD(T) step of each composite approach on 16 physical cores on a node equipped with 26-core Intel Ice Lake CPUs (2.20 GHz) for selected species of the W4-11 dataset.. S5

Table S4. Post-CCSD(T) step time (% of total wall time) for each composite approach with timing data from Table S3.. S7

Complete references.. S8

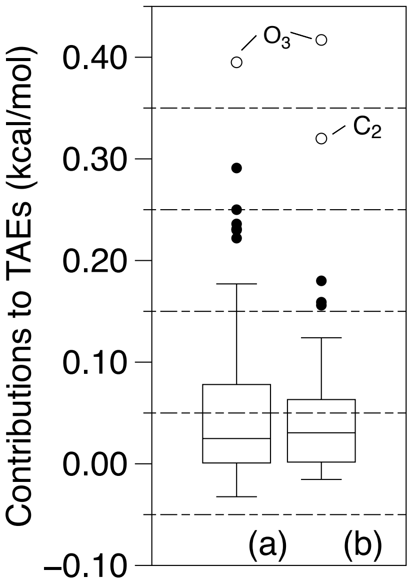

Figure S1. Box plots of CCSDTQ5 – CCSDTQ contributions to TAEs using (a) VDZ(p,s) for the W4-08 dataset; (b) VDZ(d,s) for the 'W4.3' subset.

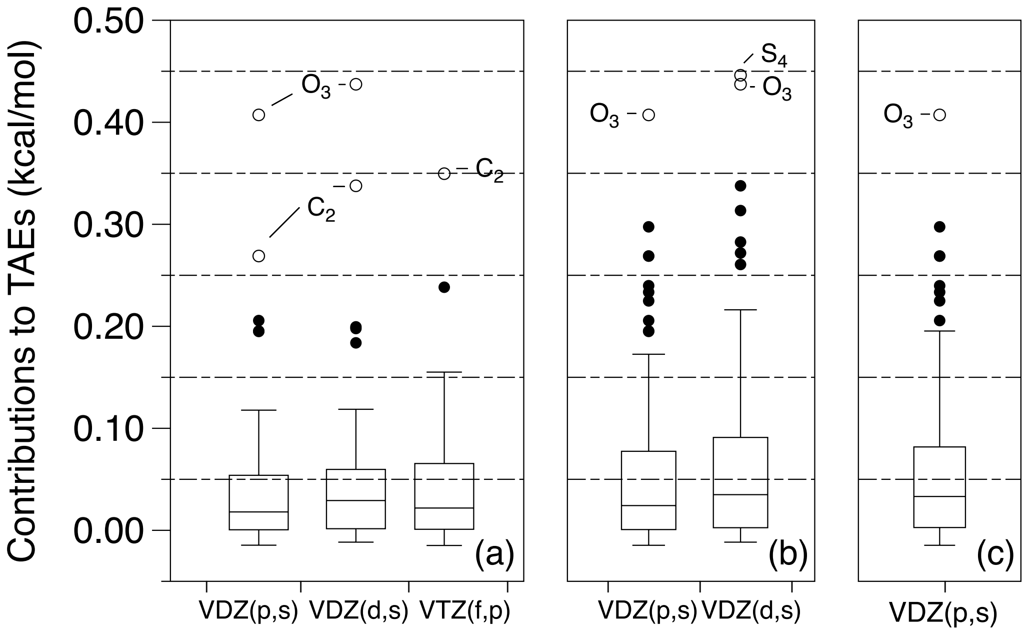

Figure S2. Box plots of CCSDTQ(5)$_\Lambda$ – CCSDTQ contributions to TAEs for (a) the 'W4.3' subset; (b) the W4-08 dataset; and (c) the W4-11 dataset.

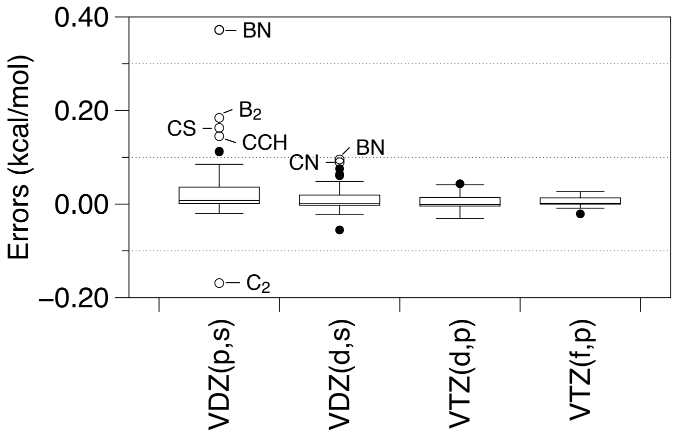

Figure S3. Box plots of CCSDTQ – CCSDT(Q) errors for TAEs relative to [CCSDTQ – CCSDT(Q)]/VQZ(g,d) for the ‘W4.3’ subset.

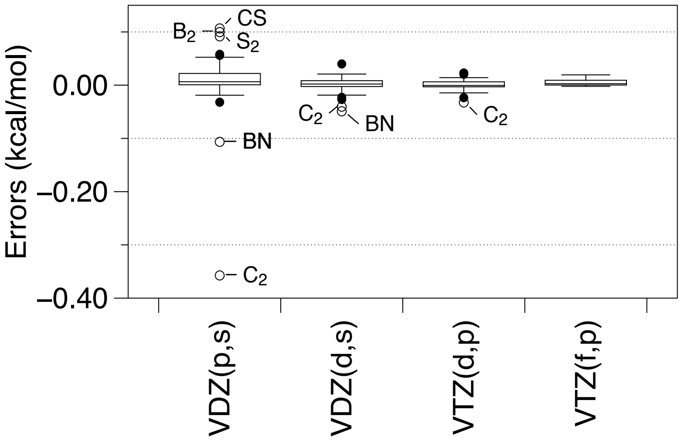

Figure S4. Box plots of CCSDTQ – CCSDT(Q)$_\Lambda$ errors for TAEs relative to [CCSDTQ – CCSDT(Q)$_\Lambda$]/VQZ(g,d) for the ‘W4.3’ subset.

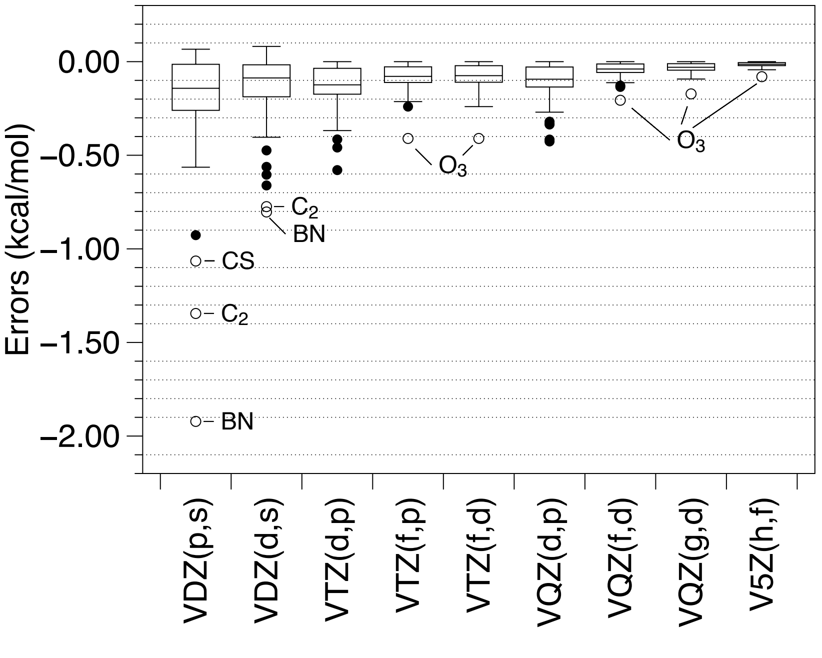

Figure S5. Box plots of CCSDT(Q) – CCSDT errors for TAEs relative to [CCSDT(Q) – CCSDT]/V{Q(g,d),5(h,f)}Z for the 'W4.3' subset.

Table S1: Relative errors (kcal/mol) of $[\hat{T}_4 - (Q)]$ and $[\hat{T}_4 - (Q)_\Lambda]$ compared to $[\hat{T}_4 - (Q)]$/VQZ(g,d) and $[\hat{T}_4 - (Q)_\Lambda]$/VQZ(g,d), respectively, for selected species of the 'W4.3' subset.

$[\hat{T}_4 - (Q)]$ vs. $[\hat{T}_4 - (Q)]$/VQZ(g,d)				
Species	VDZ(p,s)	VDZ(d,s)	VTZ(d,p)	VTZ(f,p)
CN	0.373	0.089	0.043	0.026
BN	0.372	0.095	-0.029	0.026
B_2	0.184	0.076	0.041	0.022
CS	0.163	0.029	0.005	0.014
CCH	0.145	0.019	0.015	0.010
C_2	-0.169	0.061	0.009	0.026
AlF	-0.021	-0.022	-0.014	-0.009
N_2	-0.011	0.009	0.023	0.015
CH_4	-0.004	-0.006	-0.001	0.000
BF	-0.003	-0.008	-0.008	-0.004

$[\hat{T}_4 - (Q)_\Lambda]$ vs. $[\hat{T}_4 - (Q)_\Lambda]$/VQZ(g,d)				
species	VDZ(p,s)	VDZ(d,s)	VTZ(d,p)	VTZ(f,p)
CS	0.107	0.021	0.007	0.015
B_2	0.100	0.007	0.014	0.010
S_2	0.092	0.018	0.004	0.014
CCH	0.058	-0.019	0.002	0.004
CN	0.056	-0.023	0.008	0.009
C_2	-0.357	-0.040	-0.032	0.007
BN	-0.106	-0.049	-0.024	0.003
BN ($^3\Pi$)	-0.032	-0.013	-0.005	0.001
P_2	-0.019	0.040	0.023	0.019
AlF	-0.013	-0.008	-0.006	-0.002

Table S2. Wall clock times (min) of composite approaches on 16 physical cores of a node equipped with dual 26-core Intel Ice Lake CPUs (2.20 GHz) for selected species of the W4-11 dataset.

	HCN	CO_2	N_2O	O_3	NCCN
W4.3	255.5	2762.1	6074.0	17943.2	31341.4
W4Λ	6.3	51.7	67.9	114.0	325.0
W4	8.5	101.0	157.5	414.7	975.7
W4.3Λ	128.2	651.8	1262.9	2214.2	3934.3
W4.4Λ	199.7	1393.7	2480.1	4201.6	10242.3
W4Lite	2.7	10.2	22.3	41.5	43.5
W4Lite,Λ	3.1	13.3	26.2	48.7	51.8

Table S3. Wall clock times (min) per post-CCSD(T) step of each composite approach on 16 physical cores on a node equipped with 26-core Intel Ice Lake CPUs (2.20 GHz) for selected species of the W4-11 dataset.

W4Λ

Component	Basis	HCN	CO_2	N_2O	O_3	NCCN
CCSDT(Q)	VTZ(f,p)	4.7	38.5	45.3	75.4	225.6
CCSDT(Q)$_Λ$	VDZ(d,s)	0.5	4.0	5.3	9.8	12.7
CCSDTQ(5)$_Λ$	VDZ(p,s)	1.1	9.1	17.2	28.8	86.7
total wall clock time		6.3	51.7	67.9	114.0	325.0

standard W4

Component	Basis	HCN	CO_2	N_2O	O_3	NCCN
CCSDT(Q)	VTZ(f,p)	4.7	38.5	45.3	75.4	225.6
CCSDTQ	VDZ(d,s)	2.4	30.2	54.7	176.0	316.6
CCSDTQ5	VDZ(p,s)	1.4	32.2	57.5	163.3	433.4
total wall clock time		8.5	101.0	157.5	414.7	975.7

W4.3Λ

Component	Basis	HCN	CO_2	N_2O	O_3	NCCN
CCSDTQ(5)$_Λ$	VDZ(d,s)	13.6	157.3	287.0	585.3	1428.7
CCSDT(Q)	VQZ(f,d)	43.5	216.0	337.5	534.2	1188.8
CCSDT	VQZ(g,d)	30.9	87.4	228.1	367.6	359.0
CCSDT(Q)	VTZ(d,p)	1.9	9.2	13.9	23.0	43.6
CCSDT(Q)$_Λ$	VTZ(f,p)	11.8	65.0	113.3	200.5	329.6
CCSDT(Val)	cc-pCVTZ(f,p)	8.3	33.6	79.3	148.4	148.2
CCSDT(+CV)	cc-pCVTZ(f,p)	18.4	83.3	203.7	355.1	436.4
total wall clock time		128.2	651.8	1262.9	2214.2	3934.3

Standard W4.3

Component	Basis	HCN	CO_2	N_2O	O_3	NCCN
CCSDTQ56	VDZ(p,s)	6.9	454.8	950.7	1681.8	15076.3
CCSDTQ	VTZ(f,p)	134.5	1691.4	3957.0	14493.1	12906.6
CCSDT(Q)	VQZ(g,d)	87.4	499.0	883.2	1264.7	2773.9
CCSDT(Val)	cc-pCVTZ(f,p)	8.3	33.6	79.3	148.4	148.2
CCSDT(+CV)	cc-pCVTZ(f,p)	18.4	83.3	203.7	355.1	436.4
total wall clock time		255.5	2762.1	6074.0	17943.2	31341.4

Table S3 (continued)

W4.4Λ						
Component	Basis	HCN	CO_2	N_2O	O_3	NCCN
comp. W4.3Λ		128.2	651.8	1262.9	2214.2	3934.3
CCSDTQ5(6)$_Λ$	VDZ(p,s)	7.5	171.3	334.3	600.7	2851.2
CCSDT(Q)(Val)	cc-pCVTZ(f,p)	18.8	163.4	242.6	402.2	851.8
CCSDT(Q)(+CV)	cc-pCVTZ(f,p)	45.1	407.2	640.4	984.4	2605.1
total wall clock time		199.7	1393.7	2480.1	4201.6	10242.3

W4Lite,Λ						
Component	Basis	HCN	CO_2	N_2O	O_3	NCCN
CCSDT	VTZ(f,p)	2.6	9.3	20.9	38.9	39.2
CCSDT(Q)$_Λ$	VDZ(d,s)	0.5	4.0	5.3	9.8	12.7
total wall clock time		3.1	13.3	26.2	48.7	51.8

Standard W4Lite						
Component	Basis	HCN	CO_2	N_2O	O_3	NCCN
CCSDT	VTZ(f,p)	2.6	9.3	20.9	38.9	39.2
CCSDT(Q)	VDZ(d,s)	0.1	0.9	1.4	2.6	4.3
total wall clock time		2.7	10.2	22.3	41.5	43.5

Table S4. Post-CCSD(T) step time (% of total wall time) for each composite approach with timing data from Table S3.

W4Λ

Component	Basis	HCN	CO_2	N_2O	O_3	NCCN
CCSDT(Q)	VTZ(f,p)	74.5	74.6	66.8	66.1	69.4
CCSDT(Q)$_\Lambda$	VDZ(d,s)	8.3	7.8	7.9	8.6	3.9
CCSDTQ(5)$_\Lambda$	VDZ(p,s)	17.1	17.6	25.4	25.3	26.7

Standard W4

Component	Basis	HCN	CO_2	N_2O	O_3	NCCN
CCSDT(Q)	VTZ(f,p)	55.4	38.1	28.8	18.2	23.1
CCSDTQ	VDZ(d,s)	28.0	29.9	34.7	42.4	32.5
CCSDTQ5	VDZ(p,s)	16.6	31.9	36.5	39.4	44.4

W4.3Λ

Component	Basis	HCN	CO_2	N_2O	O_3	NCCN
CCSDTQ(5)$_\Lambda$	VDZ(d,s)	10.6	24.1	22.7	26.4	36.3
CCSDT(Q)	VQZ(f,d)	33.9	33.1	26.7	24.1	30.2
CCSDT	VQZ(g,d)	24.1	13.4	18.1	16.6	9.1
CCSDT(Q)	VTZ(d,p)	1.5	1.4	1.1	1.0	1.1
CCSDT(Q)$_\Lambda$	VTZ(f,p)	9.2	10.0	9.0	9.1	8.4
CCSDT(Val)	cc-pCVTZ(f,p)	6.5	5.2	6.3	6.7	3.8
CCSDT(+CV)	cc-pCVTZ(f,p)	14.3	12.8	16.1	16.0	11.1

Standard W4.3

Component	Basis	HCN	CO_2	N_2O	O_3	NCCN
CCSDTQ56	VDZ(p,s)	2.7	16.5	15.7	9.4	48.1
CCSDTQ	VTZ(f,p)	52.7	61.2	65.1	80.8	41.2
CCSDT(Q)	VQZ(g,d)	34.2	18.1	14.5	7.0	8.9
CCSDT(Val)	cc-pCVTZ(f,p)	3.2	1.2	1.3	0.8	0.5
CCSDT(+CV)	cc-pCVTZ(f,p)	7.2	3.0	3.4	2.0	1.4

W4.4Λ

Component	Basis	HCN	CO2	N_2O	O_3	NCCN
	comp. W4.3Λ	64.2	46.8	50.9	52.7	38.4
CCSDTQ5(6)$_\Lambda$	VDZ(p,s)	3.8	12.3	13.5	14.3	27.8
CCSDT(Q)(Val)	cc-pCVTZ(f,p)	9.4	11.7	9.8	9.6	8.3
CCSDT(Q)(+CV)	cc-pCVTZ(f,p)	22.6	29.2	25.8	23.4	25.4

Table S4. (continued)

W4Lite,Λ						
Component	Basis	HCN	CO_2	N_2O	O_3	NCCN
CCSDT	VTZ(f,p)	83.0	69.7	79.7	79.9	75.6
$CCSDT(Q)_\Lambda$	VDZ(d,s)	17.0	30.3	20.3	20.1	24.4

Standard W4Lite						
Component	Basis	HCN	CO_2	N_2O	O_3	NCCN
CCSDT	VTZ(f,p)	94.8	90.9	93.6	93.8	90.0
CCSDT(Q)	VDZ(d,s)	5.2	9.1	6.4	6.2	10.0

Complete references

(43) Kállay, M.; Nagy, P. R.; Mester, D.; Rolik, Z.; Samu, G.; Csontos, J.; Csóka, J.; Szabó, P. B.; Gyevi-Nagy, L.; Hégely, B.; Ladjánszki, I.; Szegedy, L.; Ladóczki, B.; Petrov, K.; Farkas, M.; Mezei, P. D.; Ganyecz, Á. The MRCC Program System: Accurate Quantum Chemistry from Water to Proteins. *J Chem Phys* **2020**, *152* (7), 074107. https://doi.org/10.1063/1.5142048.

(62) Frisch, M. J.; Trucks, G. W.; Schlegel, H. B.; Scuseria, G. E.; Robb, M. A.; Cheeseman, J. R.; Scalmani, G.; Barone, V.; Petersson, G. A.; Nakatsuji, H.; Li, X.; Caricato, M.; Marenich, A. V.; Bloino, J.; Janesko, B. G.; Gomperts, R.; Mennucci, B.; Hratchian, H. P.; Ortiz, J. V.; Izmaylov, A. F.; Sonnenberg, J. L.; Williams-Young, D.; Ding, F.; Lipparini, F.; Egidi, F.; Goings, J.; Peng, B.; Petrone, A.; Henderson, T.; Ranasinghe, D.; Zakrzewski, V. G.; Gao, J.; Rega, N.; Zheng, G.; Liang, W.; Hada, M.; Ehara, M.; Toyota, K.; Fukuda, R.; Hasegawa, J.; Ishida, M.; Nakajima, T.; Honda, Y.; Kitao, O.; Nakai, H.; Vreven, T.; Throssell, K.; Montgomery, J. A., Jr.; Peralta, J. E.; Ogliaro, F.; Bearpark, M. J.; Heyd, J. J.; Brothers, E. N.; Kudin, K. N.; Staroverov, V. N.; Keith, T. A.; Kobayashi, R.; Normand, J.; Raghavachari, K.; Rendell, A. P.; Burant, J. C.; Iyengar, S. S.; Tomasi, J., C.; Cossi, M.; Millam, J. M.; Klene, M.; Adamo, C.; Cammi, R.; Ochterski, J. W.; Martin, R. L.; Morokuma, K.; Farkas, O.; Foresman, J. B.; Fox, D. J. Gaussian 16, Rev. C.01. *Gaussian, Inc., Wallingford, CT*. 2016.

(63) Werner, H.-J.; Knowles, P. J.; Manby, F. R.; Black, J. A.; Doll, K.; Heßelmann, A.; Kats, D.; Köhn, A.; Korona, T.; Kreplin, D. A.; Ma, Q.; Miller, T. F.; Mitrushchenkov, A.; Peterson, K. A.; Polyak, I.; Rauhut, G.; Sibaev, M. The Molpro Quantum Chemistry Package. *J Chem Phys* **2020**, *152* (14), 144107. https://doi.org/10.1063/5.0005081.
